# Grand Challenge in Psychopharmacology: Setting Priorities to Shape a Bright Future

**DOI:** 10.3389/fpsyt.2017.00015

**Published:** 2017-02-10

**Authors:** Roberto Ciccocioppo

**Affiliations:** ^1^School of Pharmacy, Pharmacology Unit, University of Camerino, Camerino, Italy

**Keywords:** history, perspective, psychiatry, neuropsychopharmacology, future orientation

## The Origin of Psychopharmacology

Psychopharmacology combines two terms, psychology and pharmacology describing how biologically active compounds including drugs regulate psychobiological processes and affect behaviors. Reinhard Lorichius originated the term in 1548 in his “Psychopharmakon, hoc est medicina animae” a collection of prayers in which the term “psychopharmakon” relates to a spiritual medicine to alleviate affliction and miserable situations of life (1, 2). Foundations of modern psychopharmacology appear in the nineteenth century with the discovery of chemical agents, such as morphine, bromide salts, chloral hydrate, and other general anesthetics. A few decades later, the psychostimulant cocaine and depressant barbiturates were identified. During the first half of the twentieth century hallucinogenic compounds such as mescaline and LSD, and psychoactive amphetamines were also discovered (2–5).

## The Magic Years of Psychopharmacology

The major breakthrough in the field came between 1950 and 1960 with the discovery of the first antipsychotic chlorpromazine, followed by MAO inhibitors and tricyclic antidepressants and a few years later by the first benzodiazepine, chlordiazepoxide. Over the next two to three decades, the field flourished and psychopharmacology became an extremely popular research area upon which interests of pharmacologists, psychiatrists, and psychologists converged. With further development in the field, the possibility of treating psychiatric conditions improved enormously, and clinical practices in psychiatry were revolutionized. The two major therapies at the time used to treat schizophrenia and affective disorders, insulin-induced hypoglycemic coma, and electroconvulsive shock were replaced by antipsychotic and antidepressant drugs. Agitation and violence often tackled with seclusion and restraint were successfully treated with barbiturates and later with benzodiazepines (3, 5, 6).

With the discovery of new molecules and mechanisms of drug actions, more sophisticated, pharmacocentric based, psychobiological theories emerged to explain psychiatric illness or to explain complex behaviors paving the way for modern psychopharmacology.

## Modern Psychopharmacology

Over the last three decades, few major scientific breakthroughs dramatically changed the field. The advent of new molecular technologies affected major research areas in biomedical sciences including psychopharmacology. New potent tools appeared enabling enquiry into the mechanisms of diseases. New hypotheses based on molecular information challenged the pharmacocentric approach used in psychiatry to determine the causes of specific disorders. At the beginning of the 1980s, gene targeting approaches arose allowing the possibility of selectively introducing or deleting genes in model animals, offering new methods to explore the genetic basis of psychiatric illness (7–11). Continued development over the years occurred *via* gene-targeting approaches. Recombinase-based techniques allowed the generation of cell/tissue/brain regions for specific knockout and knockin mouse lines, offering unprecedented possibility to explore brain functions (10). The recent introduction of CRISPR-Cas-based techniques now makes gene editing easier, potentially allowing genome editing at multiple sites not only in model animals but also in other species, including humans (12–14).

During early 2000s, the human genome was sequenced bringing new expectations of deciphering the genetic basis of mental illness (15, 16). Psychiatry was revolutionized again by the development of high-throughput genotyping platforms permitting genetic association studies and identifying genetic risk factors for psychiatric disorders (17, 18). Psychiatry genetics and genome-wide association studies could help to identify new drug targets and potentially new pharmacotreatments for brain disorders (19).

The development of epigenetics represents a third important breakthrough (20). For decades, the “nature vs nurture” debate played a significant role in behavioral sciences particularly in the field of psychiatry and psychopharmacology. If on the one hand preclinical and clinical research demonstrated that the presence of specific genetic traits contributes to the development of affective disorders and psychiatric conditions. On the other hand, it was also clear that the process of actually developing the disease was influenced by a number of other elements generically defined as “environmental factors.” Such factors include stress, life style, education, individual experiences, and several more (21). With the development of epigenetics, the “nature vs nurture” dichotomy is dissipating. It is now clear that the environment can influence the expression of genetic traits and that genome and epigenome interact with each other to shape endophenotypes (21, 22). Despite genetic vulnerability, the environment influences the propensity to develop a disease. On the other hand, genetically non-vulnerable subjects, if exposed to particularly detrimental environments or to certain individual experiences can develop psychiatric pathologies.

Optogenetics and chemogenetics are two other recent innovations heavily influencing the field of psychopharmacology (23–26). These new methods allow the insertion of light or drug-sensitive artificial receptors into specific subset of neurons (27). These new approaches, combined with brain imaging techniques, electrophysiology, and pharmacology, allow unprecedented possibilities to explore neuronal circuitry, cytoarchitectures, and functions. Optical control of specific cell type on a millisecond timescale during animal behavior helps to decipher the contribution of specific neuronal circuitry and cellular mechanisms in the organization of emotional states, cognitive processes, and complex behaviors in general. For instance, optogenetics utilized *in vivo* helps unravel physiological mechanisms associated with specific forms of learning and memory, motivation and reward, in conjunction with the regulation of the sleep-wake cycle, feeding, etc. (27). Optogenetics has also helped to link dysfunctions of specific neurocircuitry or neural ensembles with negative emotions and psychiatric conditions, such as fear, anxiety, depression, and addiction (28, 29). It is envisioned that future refinement of optogenetic techniques will open the possibility to use light to treat neurological and psychiatric conditions (23).

## Future Steps in Psychopharmacology

Breakthrough of genetics and molecular biology in psychopharmacology contributed enormously to understanding the etiopathological basis of mental illness and helped to identify several new targets for drug development. Nevertheless, it has been disappointing to observe that despite these advancements, successful introductions of new medications into clinical practice remain very modest.

Major pharmaceutical companies are currently disengaging from research and drug-discovery programs in psychiatry, and over the last 10 years, very few new products have seen the market (19, 30). Most of the treatments currently available remain or are derived from those agents introduced by serendipitous discoveries during the mid 50s; the magic years of psychopharmacology. Whereas very few innovative biological mechanisms identified over the last 30 years have been successfully targeted with new medications. The reasons for this lack of success are multiple. On the one hand, development of CNS drugs is intrinsically complicated, and compared to the treatment of other disease areas, success rates remain low. Moreover, for neuropsychiatric drugs, the stringent requirements imposed by regulatory agencies represent a disincentive for pharmaceutical industries. Paradoxically, a third reason hampering drug development in neuropsychiatry is the attraction that molecular biology and genetics had for basic scientists. This shift of interest has drawn researchers’ interests toward these new disciplines draining human and financial resources from classical neuropsychopharmacology. Genetic and molecular biology techniques progressively replaced classical pharmacological approaches to probe the validity of biological targets or to test new disease etiopathological mechanisms. There is general optimism that introduction of these new approaches will help to advance our knowledge in psychiatry and to develop more efficacious treatments in the long run. In the short term, reduction of interest in using pharmacological tools to investigate biomedical questions is reducing the cross-fertilization between basic science and applied research in pharmaceutical industry.

Actions should be taken to engage basic science and pharmaceutical industry in a common effort to increase the chances of successful development of new medications. The development of a clear and well-structured translational approach for medication development is a major challenge in CNS drug development. From the preclinical standpoint, it is essential to develop better animal models to mimic the complex traits typical of psychiatric conditions. At the clinical level, when possible, it is important to deconstruct psychiatric categories into identifiable endophenotypes and to cluster patients into populations as homogeneous as possible (31). In addition, it is important to have biomarkers or surrogate markers that can be determined in laboratory animals and in humans. Finally, pharmacological manipulations should be used in conjunction with molecular and genetic approaches.

Gender difference in psychopharmacology is another aspect that needs attention. In 1993, the FDA issued its Guideline for the Study and Evaluation of Gender Differences in the Clinical Evaluation of Drugs. From the publication of this document, the inclusion of female subject in clinical trials increased progressively. Nevertheless, preclinical research is still based on animal models originally developed using males. Traditionally, males are preferred to females to avoid potential confounding factors linked, for example, to hormonal fluctuations associated with the estrus cycle. The perception is that these fluctuations can influence behavior, decrease the homogeneity of results, and enhance the variability in response to drugs (32). It is shortsighted to ignore women who represent more than half of the population at global level, who use such medications at higher rate, and who show differences from males in etiopathology and prevalence. For instance, it is well known that mental illnesses, such as depression, anxiety, eating disorders, and post-traumatic stress disorder, show higher prevalence in females, whereas alcohol abuse and antisocial personalities predominate in males (33, 34).

Male and female also differ in drug response due to several pharmacokinetic (PK) and pharmacodynamic (PD) factors (35). Hence, inclusion of females in preclinical tests is important to better determine the PK/PD parameters predictive of drug response in clinical populations. The Zolpidem case provides a prototypical example of sex-related difference in response to a drug. This hypnotic compound is subjected to a lower metabolic elimination rate in females compared to males. This has inspired a historical decision by FDA in 2013 to cut the recommended therapeutic dose in half for women. This is the first example ever of an approved medication explicitly taking into account gender-related differences in drug response. Establishment of gender oriented policies in drug development is now a priority, and more is expected in the near future.

Notably, neuroscience is a discipline in which male bias in preclinical research is the norm (36). This gap needs to be filled, and better animal models are needed, taking into account sex-related differences in behavior and in response to drugs. Awareness that gender differences exist in neuropsychiatric disease, etiopathology, and in response to treatment intervention is critical to enhance more efficacious and safer medications for mental illness.

Exploration of disease etiopathology and identification of new targets amenable to drug development including genetic, epigenetic, brain imaging, proteomics, and behavioral and pharmacological data should occur altogether. The need to build large databases containing all this information is pressing. The computational and the statistical tools to acquire “big data” in biomedical science are now becoming available and are relatively user friendly. If made open source these databases would be of inestimable value for investigators involved in basic science as well as in applied research. There are several advantages in making scientific data open. For research teams, data sharing is a unique opportunity to synergize and enhance the possibility to successfully achieve ambitious scientific discoveries. It is a cost-effective strategy, especially when expensive technologies are applied to generate the data. Moreover, for those investigators that do not have access to expensive technologies, open data enable the possibility to indirectly benefit from them. This can be a unique instrument to help cultivating high quality research also in those countries that do not or cannot invest enough money in expensive technologies. Ultimately, open data programs can help to raise the research impact at global level thus helping to fill the gap between high and low developed countries. Open data also help to evaluate the quality of the research, contribute to prevent its duplication, and promote reproducibility.

In the promotion of open data policies, there are also obstacles to surmount. These include issues arising from intellectual property rights and the inclination of researchers to protect their data. Nevertheless, data sharing approaches have proven successful with the Human Genome Project, and there is a lot of expectation from the Brain initiative and the European Human Brain project. Learning from these experiences provides a substantial help to advance the field of psychopharmacology.

Our understanding of brain function, cognition, emotion, and mental illness is advancing rapidly. In a common effort, basic and applied preclinical and clinical research should concentrate energy toward the validation of new pharmacological targets and improve the success rate in drug development in psychiatry.

## Author Contributions

The author confirms being the sole contributor of this work and approved it for publication.

## Conflict of Interest Statement

The author declares that the research was conducted in the absence of any commercial or financial relationships that could be construed as a potential conflict of interest.
